# Editorial: Medicinal Plants for Cardiovascular and Neurodegenerative Aging-Related Diseases: From Bench to Bedside

**DOI:** 10.3389/fphar.2020.585155

**Published:** 2020-09-23

**Authors:** Yue Liu, Valentina Echeverria, Youhua Xu

**Affiliations:** ^1^Cardiovascular Disease Centre, Xiyuan Hospital of China Academy of Chinese Medical Sciences, Beijing, China; ^2^Cardiovascular Disease Centre, National Clinical Research Center for Chinese Medicine Cardiology, Beijing, China; ^3^Laboratorio de Neurobiología, Facultad de Ciencias de la Salud, Universidad San Sebastián, Concepción, Chile; ^4^Research and Development Department, Bay Pines VA Healthcare System, Bay Pines, FL, United States; ^5^State Key Laboratory of Quality Research in Chinese Medicines, Macau University of Science and Technology, Macau, Macau

**Keywords:** medicinal plants, aging, age-related diseases, cardiovascular disease, neurodegenerative diseases

Aging is a progressive and multi-step degeneration in the physiological functions and metabolic processes of living organisms until death. It represents the main risk factors for a number of debilitating diseases and contributes to increase in mortality (Ahadi et al., 2020). With increasing life expectancy, the number of patients with aging related diseases will continue to rise, leading to an increased healthcare burden. There is a need for new therapies to treat this growing number of patients in a manner that is effective and sustainable (Liu and Chen, 2012). Aging presents profound physiological changes in the cardiovascular system and the relationship between cardiovascular pathology and neurodegenerative diseases is well known (Luo et al., 2020). Ischemic events due to cardiac pathology or stroke can lead to cardiovascular dementia and the development of Alzheimer’s disease (Daniele et al., 2020). Cardiac pathology has also been found to be associated with other neurodegenerative conditions such as Huntington’s disease (Critchley et al., 2018). Interestingly, it is hypothesized that factors systemically linking these pathologies are associated with neuroinflammation and oxidative stress pathways (Liu et al., 2019; Tian et al., 2019). While the underlying mechanisms in the aging heart or brain are still unclear, greater research is needed to test the potential for pharmacological interventions in these pathways.

Natural plants have been associated with traditional medical approaches for thousands of years all over the world. Great varieties of plants have persisted in such usage for medicinal treatments in various cultures and many new drugs have been discovered from herbal sources (Liu et al., 2013; Liu and Liu, 2020). The 2015 Nobel Prize awarded to Professor Tu, a Chinese pharmaceutical scientist, for the discovery of artemisinin renewed global interest in herbal medicine, and potential integration of such approaches into evidence-based medical systems (Tu, 2011). Medicinal plants have shown to be beneficial in decreasing the occurrence or delay of the neurodegenerative process induced by cardiovascular and mixed pathological events. It is believed that many of the medicinal herbs have anti-aging properties (Zhao et al., 2020), but the mechanisms and safety remain unclear.

This Research Topic is a collection of nineteen articles adapting a critical approach designed to robustly test the clinical effects, mechanisms and safety of adequately-characterized herbal medicine and their active components for aging-related diseases, in particular, cardiovascular and neurodegenerative diseases.

Alzheimer’s disease (AD) is the most common neurodegenerative disorder associated with aging that causes memory, thinking and behavior disorders. Currently, FDA-approved anti-AD drugs (rivastigmine, galantamine, donepezil, memantine) are potential effective in the short term but are unable to halt or reverse disease progression (Dong et al., 2019). In November 2019, Sodium oligomannate received its first approval in China for the treatment of mild to moderate AD to improve cognitive function (Syed Yahiya, 2020), therapeutically remodels gut microbiota and suppresses gut bacterial amino acids-shaped neuroinflammation to inhibit AD progression (Wang et al., 2019), but there is some controversy in China (Rao, 2020; Wang et al., 2020). In this Research Topic, there are three research articles and two review articles describing the potential efficacy and related mechanisms of medicinal plants for AD or brain aging. An research article by Yang et al. evaluated the *in vitro* and *in vivo* effects of a traditional Chinese medicinal formula named *Yizhiqingxin formula* (YQF) on autophagy, the authors found that YQF could improve spatial learning in APP/PS1 mice and ameliorate the accumulation of Aβ while promoting autophagy *via* mTOR pathway modulation. Peng et al.’s article studied the molecular mechanisms of another traditional Chinese medicinal formula, *Baizhu decoction* (BZD) for AD, they found that middle- and high-doses of BZD ameliorated the behavioral aspects of 5xFAD transgenic mice in elevated plus maze, Y maze and Morris water maze tests with reduced BACE1 and PS1, resulting in a reduction of Aβ plaques and oxidative damage. Study by Wang et al. showed that β-Asarone, the main constituent of *Acorus tatarinowii Schott*, significantly dose-dependently increased cell proliferation and decreased cytotoxicity, inhibited SA-bGal and improved cell senescence in PC12 cell AD model. In a literature review, Balkrishna et al. reviewed the phytochemical profile, pharmacological attributes and medicinal information of *Convolvulus prostratus* (a nootropic herb used in traditional medicinal systems) with comprehensive research gap analysis, *Convolvulus prostratus* is mainly endowed with neuroprotective, nootropic and neuro-modulatory activities without any signs of toxicity and neurodegeneration up to a dose of 2000 mg/kg in mice. Another review article by Martínez-Coria et al. analyzed the most important pharmacological actions of *dihydromyricetin* (one of the main flavonoids of some Asian medicinal plants), including its antioxidant, anti-inflammatory and neuroprotective actions, as well as its ability to restore GABA neurotransmission, improve motor and cognitive behavior.

One research article by Fu et al. studied neuroprotective effects of *Qingnao dripping pills*(QNDP), a traditional Chinese prescription, for Ischemic stroke. The authors found that QNDP had neuroprotective effects against cerebral ischemia *via* inhibiting NLRP3 inflammasome signaling pathway, and was a potential candidate for the future treatment of ischemic stroke.

Heart failure (HF) is the terminal state of all cardiovascular diseases, and there is limited effective drug up to now (McMurray et al., 2019; Triposkiadis et al., 2019). The discovery of potential drugs for the treatment of heart failure from medicinal plants is of great clinical value. Lu et al. explore the effect of *Qishen Granule* (an effective Chinese medicine for treating heart failure) on the release of splenic monocytes, the recruitment of monocytes into heart tissues and the differentiation of macrophages under ischemic conditions. The authors reported that compounds of Chinese medicine have synergistic effects on cardiac and splenic organs through regulating differentiation of monocytes/macrophages in inhibiting myocardial remodeling. Li et al. reported that administration of Shengmai (used to treat acute and chronic heart failure in China), a traditional Chinese medicine extracted from *Panax ginseng C.A. Mey., Ophiopogon japonicus (Thunb.) Ker Gawl., and Schisandra chinensis (Turcz.) Baill*., suppressed ang II–induced cardiomyocyte hypertrophy and apoptosis *via* activation of the AMPK signaling pathway through an energy dependent mechanisms. Another TCM, *Shenfu injection* (SFI) has been widely used for the treatment of shock and HF in China. The study by Zhu et al. showed that the vasodilation effect of SFI in thoracic aorta is mediated entirely by enhancing eNOS, activity through the PI3K/Akt signaling pathway, providing novel knowledge on the effect of SFI on shock and HF for future clinical applications. *Yixinshu Capsules* (YXSC) are widely used in China for the treatment of heart failure (HF) with better clinical efficacy, but the therapeutic mechanisms are not well understood. In the study by Xu et al., a metabonomic approach based on integrated UPLC-Q/TOF-MS technique and MALDI-MS was utilized to explore potential metabolic biomarkers increasing the understanding of HF and assessing the potential mechanisms of YXSC against HF, they proposed strategy may contribute to the understanding of the complex pathogenesis of ischemia-induced HF and the potential mechanism of YXSC.

Many medicinal plants do not enter the blood after they are taken orally, growing evidence suggest the role of the gut microbiota as targets for the multifunctional role of medicinal plants for cardiovascular diseases (CVD) (Gong et al., 2020). There are three research articles focused on the changing characteristics of gut microbiota to elucidate the mechanisms of medicinal plants in the treatment of cardiovascular disease. Zhang et al. reported that long-term administration of *Naoxintong* capsule, a Chinese medicine, increased the diversity of gut microbiota, influenced the microbiome structure and composition stably, and revered the increase of the ratio of the Firmicutes to Bacteroidetes in relative abundance, inhibited the development of cardiovascular diseases by ameliorating high-fat diet-induced metabolic disorders and partly through improving gut microbiota. Wu M. et al. observe the effects of high or low doses of *berberine* on atherosclerosis and gut microbiota modulation, their study found that anti-atherosclerotic action of berberine may be partly attributed to changes in composition and functions of gut microbiota which may be associated with anti-inflammatory and metabolism of glucose and lipid. The study by Wu D. et al. provides for the first time the morphological, biochemical, and molecular evidence supporting the protective effects of *baicalin* (the major flavonoid component of *S. baicalensis Georgi*) on the intestinal integrity in the spontaneously hypertensive rats, which may help better understand the therapeutic actions of *S. baicalensis Georgi* in the treatment of hypertension.

An important feature of this Research Topic collection, many well-known scholars have contributed a lot of wonderful literature reviews about medicinal plants for aging-related cardiovascular and neurodegenerative diseases. Souza-Junior et al. discuss the ethnobotanical characteristics, phytochemical constitution, and cardiovascular and neurological properties of *A. canelilla*, systematizing the knowledge about the species and proposing new perspectives for research and development. Shaito et al. overview the data on the ethnopharmacological therapeutic potentials and medicinal properties against CVDs (myocardial infarction, hypertension, peripheral vascular diseases, coronary heart disease, cardiomyopathies, and dyslipidemias) of *Ginseng, Ginkgo biloba, Ganoderma lucidum, and Gynostemma pentaphyllum*. Michel et al. reviewed the potential use of medicinal plants from Asteraceae and Lamiaceae Plant Family in CVDs. Atherosclerosis is the main pathological mechanism of aging-related cardiovascular and neurodegenerative diseases, characterized by changes of blood lipids profile and inflammation in vessel wall. Kirichenko et al. reviewed the databases PubMed and Scopus (until November, 2019) to investigate the medicinal plants possessing anti-atherosclerotic activity in experimental and clinical studies. Wu M. et al. summarize the blood lipid lowering, anti-oxidative, anti-inflammatory, and vascular endothelial protection by hawthorn and its extracts, providing a potential use for atherosclerosis. This Research Topic also includes a systematic preclinical review of the effect of Notoginsenoside R1 (NGR1) for ischemia/reperfusion injury. The study by Tong et al. showed the organ protection effect of NGR1 after I/R injury, suggesting that NGR1 can potentially become a novel drug candidate for ischemic diseases.

From the above-mentioned 19 articles, this Research Topic provides recent evidence about efficacy, mechanisms, and safety of medicinal plants for cardiovascular and neurodegenerative aging-related diseases. We hope this topic will help to achieve a deeper understanding of the effect of medicinal plants for aging-related diseases.

## Author Contributions

All authors listed have made a substantial, direct, and intellectual contribution to the work, and approved it for publication.

## Funding

VE was supported by a Grant Fondecyt 1190264 from ANID, Chile.

## Conflict of Interest

The authors declare that the research was conducted in the absence of any commercial or financial relationships that could be construed as a potential conflict of interest.
